# Intraoperative Frozen Section Diagnosis of Giant Cell Arteritis

**DOI:** 10.7759/cureus.68222

**Published:** 2024-08-30

**Authors:** John Mallow, Moretta Nielson, Achal Jadhav, Shriya Sridhar, Trevor Killeen, Christopher J Tignanelli, Michael A Linden, Faqian Li, James V Harmon

**Affiliations:** 1 Department of Surgery, University of Minnesota, Minneapolis, USA; 2 Department of Surgery, St. John's Medical College, Bengaluru, IND; 3 Department of Laboratory Medicine and Pathology, University of Minnesota, Minneapolis, USA

**Keywords:** vasculitis, case report, frozen section, temporal artery biopsy, giant cell arteritis

## Abstract

Giant cell arteritis (GCA) is a systemic vasculitis of medium and large vessels that is diagnosed using temporal artery biopsy (TAB). In this case report, we explored the benefits of frozen section analysis as a rapid intraoperative diagnostic technique for GCA. We present the cases of two patients who underwent TAB with frozen section analysis, to demonstrate the value of this technique in initiating immediate treatment and potentially avoiding unnecessary contralateral biopsies when the frozen section of the first biopsy confirms GCA. We recommend further investigation into the use of frozen section analysis for patients suspected of having GCA, who might otherwise undergo bilateral TAB.

## Introduction

Giant-cell arteritis (GCA) can have devastating clinical effects if left untreated; however, treatment is highly effective once initiated and significantly reduces complications, such as permanent blindness. The gold standard for diagnosis of GCA is temporal artery biopsy (TAB), followed by routine hematoxylin and eosin (H&E) histological examination with elastin stain and immunohistochemical studies of lymphocyte and macrophage markers, where appropriate. Biopsies are often taken from both the left and right temporal arteries, as histological changes can differ in up to 45% of cases [1]. This has led to debate among surgeons regarding whether bilateral biopsies should be routinely performed to account for the potential discordance, or if a unilateral biopsy can suffice. More than two-thirds of surgeons typically opt for unilateral TAB, with fewer than 20% choosing bilateral TAB. Among those advocating for initial unilateral biopsy, there is disagreement regarding whether a negative initial biopsy should be followed by a contralateral biopsy; approximately half of physicians proceed with a second biopsy in this scenario [2]. Such variation in practice may result in suboptimal patient outcomes, due to the high discordance rates between findings in the left and right temporal arteries, underscoring the need for a standardized approach to TAB for GCA diagnosis, which should prioritize high diagnostic sensitivity while minimizing unnecessary surgeries. With the aim of meeting these standards, frozen section diagnosis has recently been employed in TAB, followed by permanent section diagnosis [1]. Frozen section analysis enables rapid identification of findings suggestive of GCA intraoperatively, which can be subsequently confirmed by permanent section. This approach offers dual benefits. First, it allows for rapid GCA diagnosis, facilitating immediate definitive treatment [1]. Second, frozen section analysis of a unilateral TAB can determine whether contralateral biopsy is needed; a positive frozen section result for GCA on one side suggests that contralateral biopsy can be deferred, while a negative result indicates that proceeding with contralateral biopsy is preferable.

While TAB is considered a relatively minor surgical procedure, it carries risks, such as potential facial nerve injury, bleeding, skin necrosis, and stroke, with higher risks associated with bilateral procedures [3]. Challenges in the diagnosis and treatment of GCA are compounded by the delicate balance between initiating steroid therapy to prevent permanent consequences of GCA and avoiding the adverse effects of glucocorticoid treatment before obtaining a biopsy diagnosis. Prolonged empiric steroid therapy before TAB can also increase false negative biopsy rates, due to the effects of steroid treatment [4].

Here, we present two cases of patients with GCA, highlighting the advantages of implementing the frozen section technique. In the first case, rapid steroid treatment was initiated in the operating room following a positive frozen section diagnosis and contralateral biopsy was deferred. The second case involved a negative initial frozen section diagnosis, prompting contralateral TAB, which yielded a positive result. Given the potential of the frozen section approach to facilitate prompt treatment decisions and prevent unnecessary contralateral procedures, we advocate for further examination of its use in diagnosing GCA.

## Case presentation

Surgical and histological procedures

Handheld bedside ultrasound and intraoperative Doppler were used as guides during the procedures. Temporal artery distribution was marked at the skin level before prepping and draping. Surgical excision of an arterial segment was conducted above the hairline, to avoid the facial nerve and enhance cosmetic outcomes. Open TABs were performed by the attending surgeon, with assistance from a surgical resident. For patient 1, a 1.6 cm segment of the right temporal artery was obtained and submitted for histological diagnosis. For patient 2, 1.5 and 1.3 cm segments of the right and left temporal arteries, respectively, were obtained and submitted for histological diagnosis. Frozen section analysis was conducted during surgery, with standard permanent histological examinations completed within the following 48 hours. Frozen sections were analyzed by H&E staining. In both cases, permanent section analysis confirmed the diagnoses obtained from frozen sections. Both patients were discharged with standard postoperative care instructions and followed up by telephone and clinic appointments and both expressed high satisfaction with the TAB procedure and the use of frozen section diagnosis. No complications were observed in either patient.

Cases

**Figure 1:**
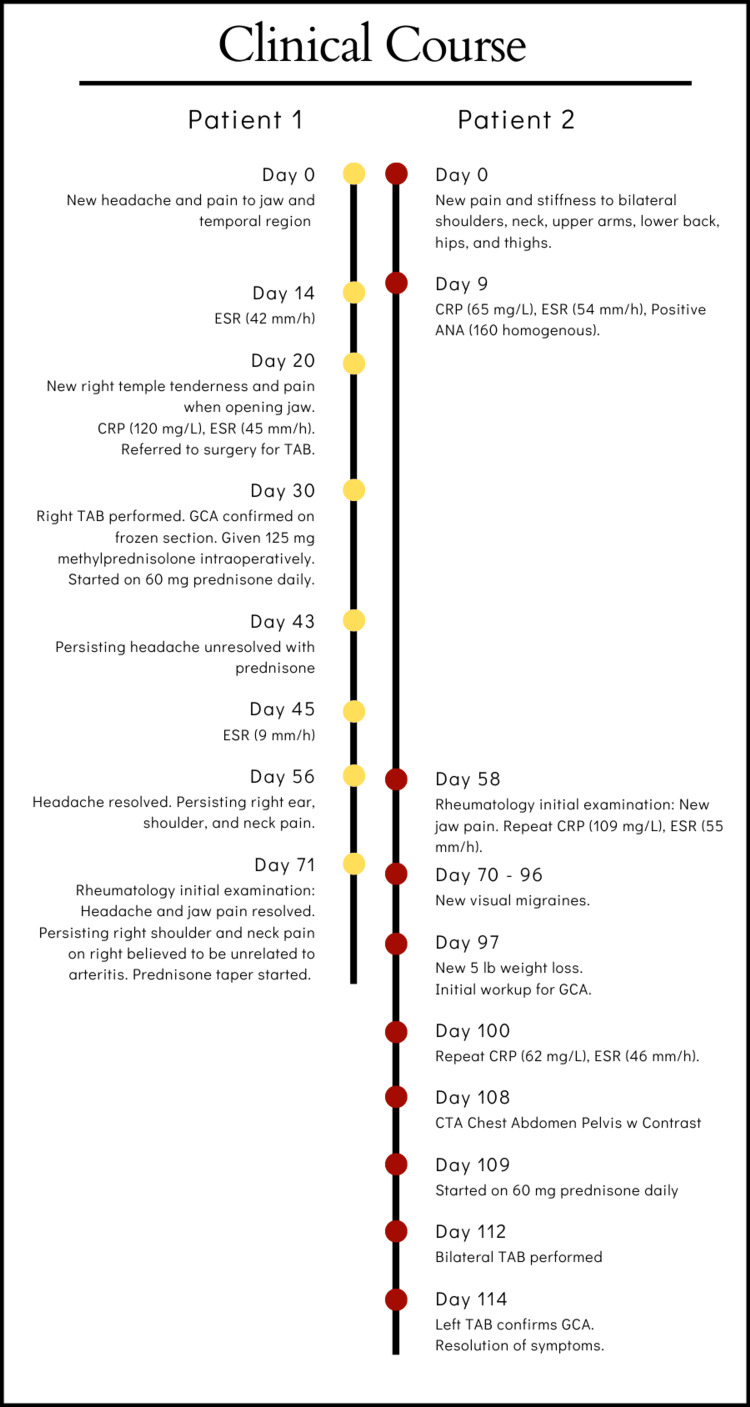
Clinical courses of patients 1 and 2. CRP: C-reactive protein, ESR: erythrocyte sedimentation rate, GCA: giant-cell arteritis, TAB: temporal artery biopsy, ANA: antinuclear antibody

A retrospective chart review of two patients who underwent TAB using frozen tissue section analysis was conducted. Written informed consent for publishing case reports was obtained from both patients. Institutional review board approval is not required for publishing case reports at our institution. Detailed clinical timelines for both patients outlining symptom onset, diagnostic results, TAB procedure, treatment initiation, and patient recovery are provided (Figure 1).

Patient 1

A 62-year-old woman with a family history of GCA presented with jaw pain, difficulty chewing, headaches, and scalp tenderness, after an initial misdiagnosis with temporomandibular joint syndrome.

Physical examination revealed a blood pressure of 175/86 mmHg, heart rate of 94 beats/min, respiratory rate of 16 breaths/min, and temperature of 36.9°C. Scalp tenderness was noted. Remaining exam results were unremarkable. Laboratory results showed an erythrocyte sedimentation rate (ESR) of 45 mm/h and a C-reactive protein (CRP) level of 120 mg/L.

**Figure 2:**
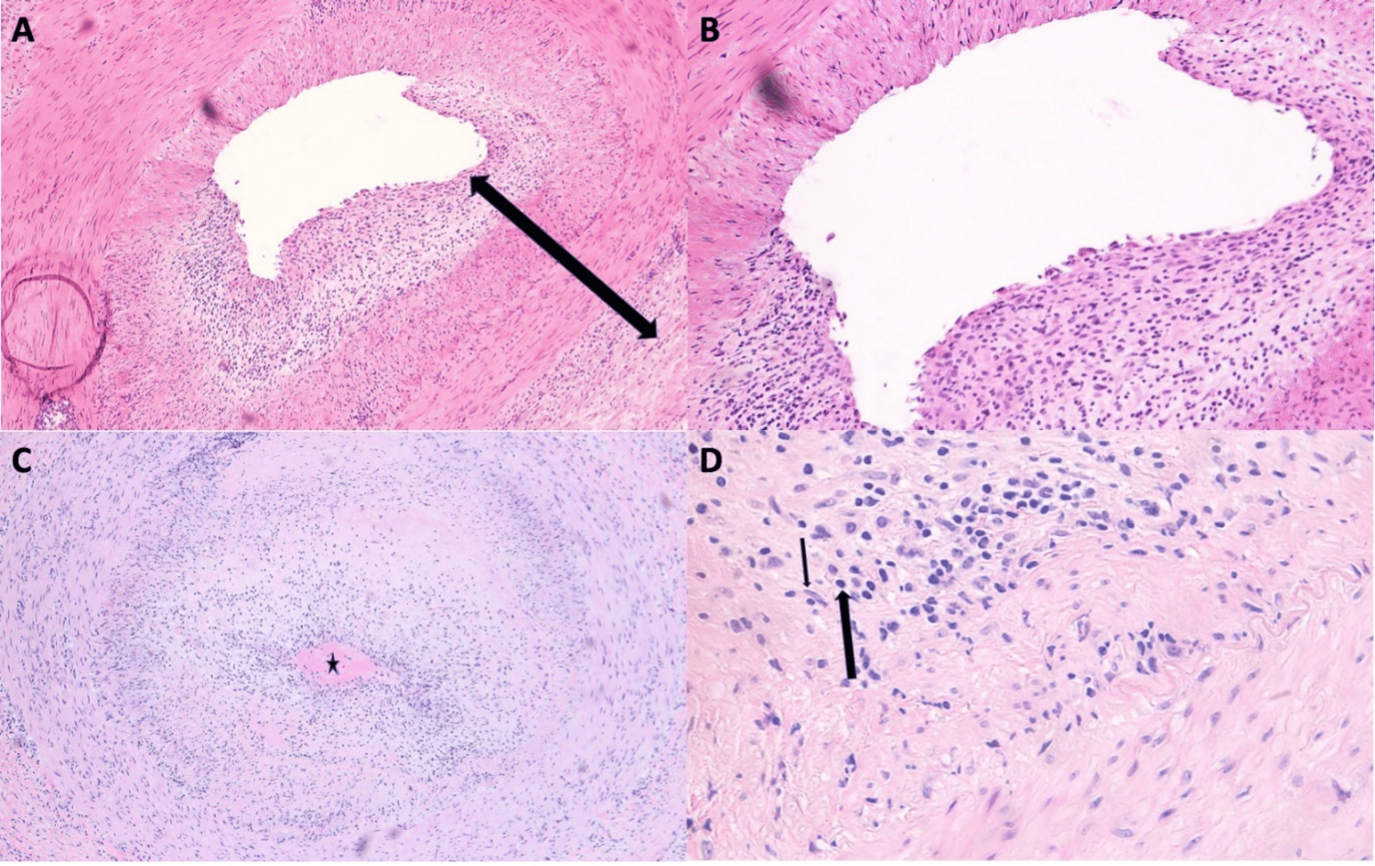
Frozen (A–B) and permanent (C–D) tissue sections of the right temporal artery of Patient 1. H&E-stained frozen sections at 10× (A) and 20× (B) magnification, exhibiting a diffuse inflammatory lymphohistiocytic infiltrate, involving the tunica intima, media, and adventitial layers (arrow transcending all three layers), consistent with inflammatory changes occurring in giant cell arteritis. H&E-stained permanent section at 10× (C) and 50× (D) magnification, exhibiting an almost occluded temporal artery (star), with prominent intimal hyperplasia and diffuse inflammatory infiltrate, comprising lymphocytes (thick arrow showing a representative cell) and epithelioid histiocytes (thin arrow showing a representative cell).

Urgent TAB of the right temporal artery was performed, during which the artery was noted to have “no arterial signal” on Doppler examination. Frozen artery section was positive for GCA, and the diagnosis was later confirmed by permanent section (Figure 2). Intravenous administration of 125 mg methylprednisolone was immediately initiated in the operating room. Postoperatively, the patient completed a short course of high-dose prednisone, followed by tapering and continued oral prednisone at 20 mg daily for three months. CRP levels decreased to 12.8 mg/L, and ESR decreased to 21 mm/h, with the patient remaining symptom-free from GCA for six years of follow-up.

Patient 2

A 66-year-old woman with a history of chronic joint pain presented with exacerbated jaw and joint pain. There was no tenderness over the temporal arteries. Her ESR level was 55 mm/h, and CRP was 109 mg/dL. Antinuclear antibody titer was 1:160, with a homogeneous pattern.

A computed tomography (CT) angiogram revealed mild wall thickening involving the thoracic aorta and the great vessels arising from the aortic arch. Mild wall thickening was also present in the abdominal aorta and femoral arteries, suggesting large-vessel vasculitis, as seen in Takayasu’s arteritis or rheumatoid arthritis. A high dose (60 mg/day) of empiric oral prednisone was initiated and urgent bilateral TAB was planned.

**Figure 3:**
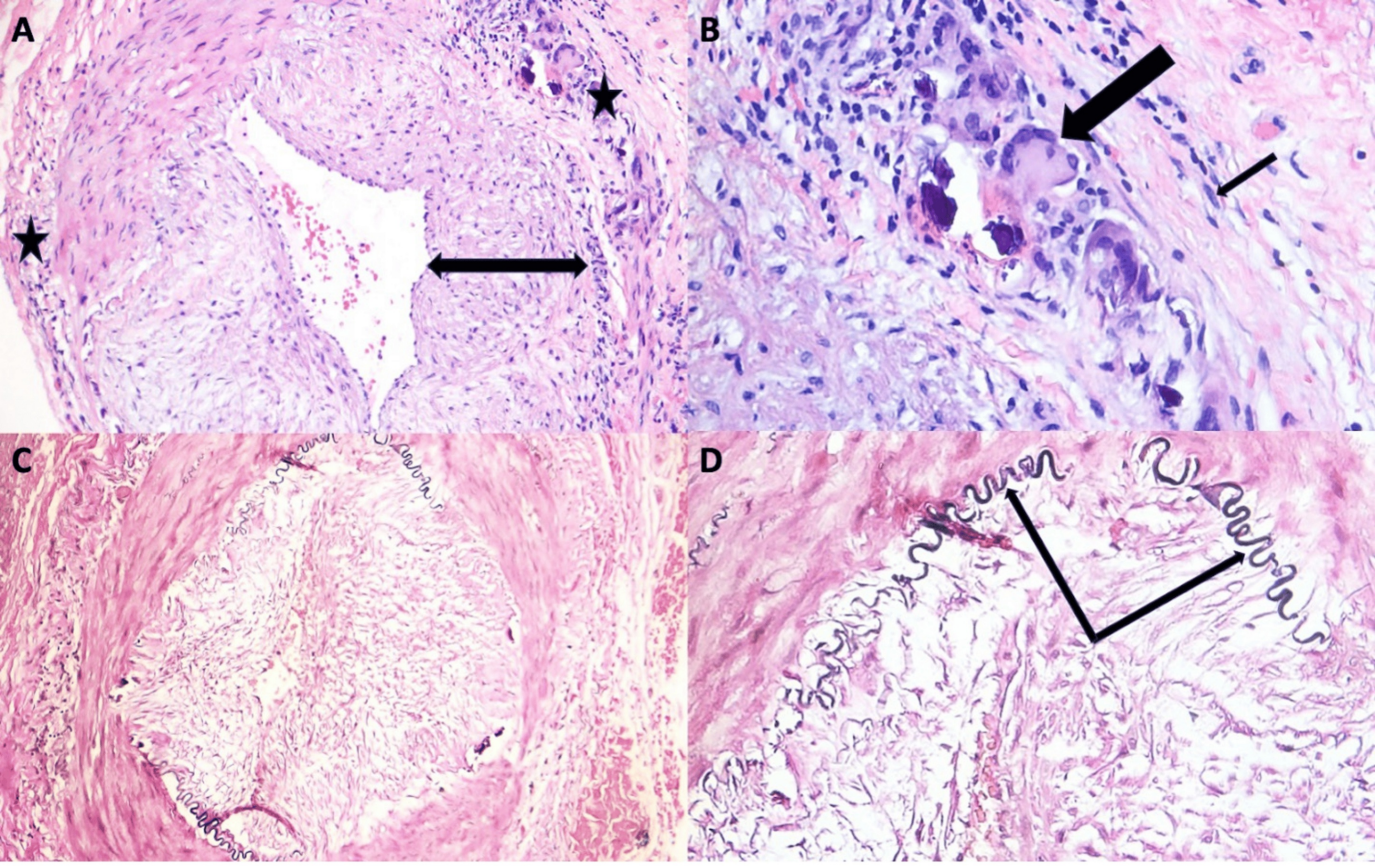
Permanent (A–D) sections of the left temporal artery of Patient 2. H&E-stained section at 20× magnification (A), exhibiting a marked thickening of the tunica intima (arrow transcending the intimal layer) with a granulomatous inflammation involving the tunica media and tunica adventitia (starred regions). H&E-stained section at 50× magnification (B), showing a multinucleated giant cell (thick arrow) and epithelioid histiocytes (thin arrow showing a representative cell). Verhoeff Van Gieson elastin staining at 20× (C) and 50× (D) magnification, showing disruption and segmental loss of the internal elastic lamina (arrows indicating segments of the internal elastic lamina).

An initial right TAB was performed and samples were obtained for both frozen and permanent sectioning. Frozen section was immediately negative for GCA, confirming the need for a contralateral biopsy. A left TAB was subsequently performed and the tissue specimen was sent for permanent pathological evaluation. Frozen section was not obtained for the left TAB as it would not have provided additional benefit to the patient. Specifically, bilateral biopsies were already performed and permanent sections would have been submitted regardless of frozen section findings. Further, empiric steroids had already been initiated. Permanent histologic examination of the right temporal artery was negative for GCA in concordance with the frozen section sample; however, histology of the left temporal artery revealed findings of GCA (Figure 3). The patient was continued on a course of oral prednisone, after which she experienced complete resolution of jaw and joint pain. Treatment was completed in less than one year, and her symptoms as well as GCA-associated laboratory findings remained normal over two years of additional follow-up.

## Discussion

GCA is a systemic vasculitis that results in destruction of medium-to-large blood vessels, particularly the thoracic aorta and extracranial arterial branches [5]. Clinical manifestations of GCA typically include jaw claudication, headache, and scalp tenderness in the area of temporal artery distribution. Approximately 20% of patients with GCA experience vision loss [6]. The etiology of GCA remains unknown, although it is likely multifactorial with involvement of both environmental and genetic factors [5,7-9]. The pathophysiology of GCA primarily involves the media of medium-to-large vessels where dendritic cells are activated through Toll-like receptors interacting with pathogen-associated molecular patterns on microbes. This activation leads to the release of chemokines and interleukins, attracting CD4+ T cells and macrophages, and ultimately resulting in granuloma formation, tissue injury, and intimal hyperplasia [10,11]. Giant cells observed in GCA are fused macrophages forming syncytia [5,12]. Certain human leukocyte antigens (HLA) I and II genotypes have been implicated as potential genetic factors associated with GCA. While testing for these genotypes may contribute to future diagnostic algorithms, current diagnostic tools include standard blood work, bedside ultrasound, radiologic studies, and biopsy [13,14].

Surgical TAB remains the gold standard for GCA diagnosis, with histological confirmation achieved in approximately 15% of biopsy samples [1]. Alternative diagnostic techniques under investigation include ultrasound examination of the temporal arteries and positron emission tomography (PET)/CT scans [15-18]; however, the quality of ultrasound results is operator-dependent and diagnostic criteria are inconsistent among systems. Additionally, PET/CT scans are expensive, involve radiation exposure, and have limited diagnostic sensitivity for GCA (approximately 70%). Consequently, TAB remains the primary diagnostic technique for GCA, facilitating prompt treatment to prevent permanent vascular complications [19].

Treatment for GCA typically involves three to 12 months of high-dose glucocorticoids followed by tapered doses, leading to symptom resolution in most cases; however, glucocorticoids may not always suppress long-term vascular complications [8,20,21]. Tocilizumab, the only approved biological agent for treating GCA, may also be considered; although, it has been associated with rare cases of leukemia and lymphoma [22,23]. Relapses occur in up to 47% of patients upon tapering or discontinuation of steroids and methotrexate or azathioprine may be considered for some of these patients [24-26]. In many cases, treatment with glucocorticoids is initiated before confirmation of a positive TAB, to prevent potential visual loss; however, this approach challenges the utility and diagnostic capability of TAB.

Given the treatment continuation in some patients, irrespective of biopsy results, the necessity of TAB has come under scrutiny [27,28]; however, a negative biopsy can mitigate long-term steroid complications; hence, TAB with permanent section analysis remains the standard procedure. While permanent section analysis boasts high sensitivity and specificity, results may not be available for several days, and discordant outcomes between right and left temporal arteries occur in up to 45% of cases, potentially leading to false-negative results from unilateral biopsies [1]. To address this, both temporal arteries are often biopsied simultaneously during initial presentation. If the frozen section analysis of the first biopsy is positive, the need for a contralateral biopsy can be obviated.

Some centers have adopted frozen section tissue analysis to expedite diagnosis before completing the TAB procedure and to avoid contralateral biopsies [1]. Permanent histologic sections are subsequently assessed to confirm the results [29]. Our dual case report highlights two specific instances where frozen section analysis was used to enhance patient outcomes. Specifically, our findings exemplify how frozen section analysis can facilitate the prompt intraoperative administration of glucocorticoids, as in the first patient. In the second patient, the initial negative frozen section diagnosis affirmed the necessity for contralateral TAB and subsequent permanent histologic evaluation confirmed the diagnosis, leading to symptom resolution; this was crucial for the second patient, who had pre-existing type I diabetes, significantly influencing her management and steroid use. These cases illustrate how our institution was able to use frozen section diagnosis to expedite treatment and avert unnecessary surgery; however, adaptability within healthcare systems must be considered.

Healthcare institutions have devised clinical care pathways for timely diagnosis and treatment. Patients suspected of having GCA may benefit from pathways emphasizing coordination and efficiency, although some institutions encounter implementation challenges [30]. Resource constraints or logistical hurdles may impede frozen section diagnosis or surgical-pathological coordination. Moreover, confidence in interpreting frozen sections varies depending on tissue preparation techniques. Despite these challenges, recent literature supports the feasibility of implementing frozen section approaches [1]. Our cases, as presented above, agree with the literature and provide two examples of how frozen section analysis can enhance patient care and minimize surgical interventions. 

Limitations

A limitation of our manuscript is its retrospective nature. Further, caution should be applied when drawing conclusions from case reports and specific inferences should not be made based on these cases alone.

## Conclusions

This case report presents two patients with GCA who benefited clinically from the use of frozen section analysis in their diagnosis. For the first patient, contralateral biopsy was avoided after intraoperative frozen section analysis confirmed GCA on the initial biopsy. The second patient required a contralateral biopsy after frozen sectioning from the initial biopsy was determined to be negative for GCA. Although a diagnosis could not be made intraoperatively, permanent sections of the contralateral biopsy were positive for GCA, allowing the care team to confidently continue corticosteroid treatment despite the patient’s history of diabetes. These two cases demonstrate the clinical benefits of frozen section analysis when applied to TAB in the diagnosis of GCA. We recommend further exploration of this technique for patients presenting with concern for GCA at medical centers where frozen section diagnosis can be reliably established.

## References

[1] Cohen DA, Chen JJ, Neth BJ (2021). Discordance rate among bilateral simultaneous and sequential temporal artery biopsies in giant cell arteritis: role of frozen sectioning based on the Mayo clinic experience. JAMA Ophthalmol.

[2] Schallhorn J, Haug SJ, Yoon MK, Porco T, Seiff SR, McCulley TJ (2013). A national survey of practice patterns: temporal artery biopsy. Ophthalmology.

[3] Mehta K, Eid M, Gangadharan A, Pritchard A, Lin CC, Goodney P, Stableford J (2022). The utility of the bilateral temporal artery biopsy for diagnosis of giant cell arteritis. J Vasc Surg.

[4] Younge BR, Cook BE Jr, Bartley GB, Hodge DO, Hunder GG (2004). Initiation of glucocorticoid therapy: before or after temporal artery biopsy?. Mayo Clin Proc.

[5] Weyand CM, Liao YJ, Goronzy JJ (2012). The immunopathology of giant cell arteritis: diagnostic and therapeutic implications. J Neuroophthalmol.

[6] Gordon LK, Levin LA (1998). Visual loss in giant cell arteritis. JAMA.

[7] Samson M, Audia S, Fraszczak J (2012). Th1 and Th17 lymphocytes expressing CD161 are implicated in giant cell arteritis and polymyalgia rheumatica pathogenesis. Arthritis Rheum.

[8] Samson M, Corbera-Bellalta M, Audia S, Planas-Rigol E, Martin L, Cid MC, Bonnotte B (2017). Recent advances in our understanding of giant cell arteritis pathogenesis. Autoimmun Rev.

[9] Akiyama M, Ohtsuki S, Berry GJ, Liang DH, Goronzy JJ, Weyand CM (2020). Innate and adaptive immunity in giant cell arteritis. Front Immunol.

[10] Rittner HL, Kaiser M, Brack A, Szweda LI, Goronzy JJ, Weyand CM (1999). Tissue-destructive macrophages in giant cell arteritis. Circ Res.

[11] Weyand CM, Goronzy JJ (2003). Medium- and large-vessel vasculitis. N Engl J Med.

[12] Brack A, Geisler A, Martinez-Taboada VM, Younge BR, Goronzy JJ, Weyand CM (1997). Giant cell vasculitis is a T cell-dependent disease. Mol Med.

[13] Carmona FD, Martín J, González-Gay MA (2015). Genetics of vasculitis. Curr Opin Rheumatol.

[14] Carmona FD, Mackie SL, Martín JE (2015). A large-scale genetic analysis reveals a strong contribution of the HLA class II region to giant cell arteritis susceptibility. Am J Hum Genet.

[15] Noumegni SR, Hoffmann C, Cornec D, Gestin S, Bressollette L, Jousse-Joulin S (2021). Temporal artery ultrasound to diagnose giant cell arteritis: a practical guide. Ultrasound Med Biol.

[16] Schmidt WA, Schäfer VS (2024). Diagnosing vasculitis with ultrasound: findings and pitfalls. Ther Adv Musculoskelet Dis.

[17] Pouncey AL, Yeldham G, Magan T, Lucenteforte E, Jaffer U, Virgili G (2024). Halo sign on temporal artery ultrasound versus temporal artery biopsy for giant cell arteritis. Cochrane Database Syst Rev.

[18] Nielsen BD, Gormsen LC (2020). 18F-fluorodeoxyglucose PET/computed tomography in the diagnosis and monitoring of giant cell arteritis. PET Clin.

[19] Thibault T, Durand-Bailloud B, Soudry-Faure A (2023). PET/CT of cranial arteries for a sensitive diagnosis of giant cell arteritis. Rheumatology (Oxford).

[20] Luo J, Wu SJ, Lacy ER (2010). Structural basis for the dual recognition of IL-12 and IL-23 by ustekinumab. J Mol Biol.

[21] Conway R, O'Neill L, O'Flynn E (2016). Ustekinumab for the treatment of refractory giant cell arteritis. Ann Rheum Dis.

[22] Villiger PM, Adler S, Kuchen S (2016). Tocilizumab for induction and maintenance of remission in giant cell arteritis: a phase 2, randomised, double-blind, placebo-controlled trial. Lancet.

[23] Stone JH, Tuckwell K, Dimonaco S (2017). Trial of tocilizumab in giant-cell arteritis. N Engl J Med.

[24] Mainbourg S, Addario A, Samson M (2020). Prevalence of giant cell arteritis relapse in patients treated with glucocorticoids: a meta-analysis. Arthritis Care Res (Hoboken).

[25] Hellmich B, Agueda A, Monti S (2020). 2018 Update of the EULAR recommendations for the management of large vessel vasculitis. Ann Rheum Dis.

[26] Mackie SL, Dejaco C, Appenzeller S (2020). British Society for Rheumatology guideline on diagnosis and treatment of giant cell arteritis. Rheumatology (Oxford).

[27] Sommer F, Spörl E, Herber R, Pillunat LE, Terai N (2019). Predictive value of positive temporal artery biopsies in patients with clinically suspected giant cell arteritis considering temporal artery ultrasound findings. Graefes Arch Clin Exp Ophthalmol.

[28] Moudrous W, Visser LH, Yilmaz T (2022). A new prediction model for giant cell arteritis in patients with new onset headache and/or visual loss. Ann Med.

[29] Butendieck R Jr, Calamia K, Sandin A (2023). A study of temporal artery biopsy for the diagnosis of giant cell arteritis. Clin Rheumatol.

[30] Lyons HS, Quick V, Sinclair AJ, Nagaraju S, Mollan SP (2020). A new era for giant cell arteritis. Eye (Lond).

